# Molecular detection of Gram-positive bacteria in the human lung through an optical fiber–based endoscope

**DOI:** 10.1007/s00259-020-05021-4

**Published:** 2020-09-11

**Authors:** Bethany Mills, Alicia Megia-Fernandez, Dominic Norberg, Sheelagh Duncan, Adam Marshall, Ahsan R. Akram, Thomas Quinn, Irene Young, Annya M. Bruce, Emma Scholefield, Gareth O. S. Williams, Nikola Krstajić, Tushar R. Choudhary, Helen E. Parker, Michael G. Tanner, Kerrianne Harrington, Harry A. C. Wood, Timothy A. Birks, Jonathan C. Knight, Christopher Haslett, Kevin Dhaliwal, Mark Bradley, Muhammed Ucuncu, James M. Stone

**Affiliations:** 1 Centre for Inflammation Research, Queen’s Medical Research Institute, University of Edinburgh, 47 Little France Crescent, Edinburgh, EH16 4TJ UK; 2 School of Chemistry, University of Edinburgh, Joseph Black Building, David Brewster Road, Edinburgh, EH9 3FJ UK; 3 The Roslin Institute and Royal (Dick) School of Veterinary Studies, University of Edinburgh, Edinburgh, UK; 4 Department of Applied Physics, Royal Institute of Technology, KTH, SE-106 91 Stockholm, Sweden; 5 Scottish Universities Physics Alliance (SUPA), Institute of Photonics and Quantum Sciences, Heriot-Watt University, Edinburgh, EH14 4AS UK; 6 Centre for Photonics and Photonic Materials, Department of Physics, University of Bath, Bath, BA2 7AY UK; 7 Department of Analytical Chemistry, Faculty of Pharmacy, Izmir Katip Celebi University, Izmir, Turkey

**Keywords:** Optical imaging, Fluorescence, Bacteria, Gram-positive, Lung, Optical endomicroscopy

## Abstract

**Purpose:**

The relentless rise in antimicrobial resistance is a major societal challenge and requires, as part of its solution, a better understanding of bacterial colonization and infection. To facilitate this, we developed a highly efficient no-wash red optical molecular imaging agent that enables the rapid, selective, and specific visualization of Gram-positive bacteria through a bespoke optical fiber–based delivery/imaging endoscopic device.

**Methods:**

We rationally designed a no-wash, red, Gram-positive-specific molecular imaging agent (Merocy-Van) based on vancomycin and an environmental merocyanine dye. We demonstrated the specificity and utility of the imaging agent in escalating in vitro and ex vivo whole human lung models (*n* = 3), utilizing a bespoke fiber–based delivery and imaging device, coupled to a wide-field, two-color endomicroscopy system.

**Results:**

The imaging agent (Merocy-Van) was specific to Gram-positive bacteria and enabled no-wash imaging of *S. aureus* within the alveolar space of whole ex vivo human lungs within 60 s of delivery into the field-of-view, using the novel imaging/delivery endomicroscopy device.

**Conclusion:**

This platform enables the rapid and specific detection of Gram-positive bacteria in the human lung.

**Electronic supplementary material:**

The online version of this article (10.1007/s00259-020-05021-4) contains supplementary material, which is available to authorized users.

## Introduction

Gram-positive bacteria are responsible for a vast range of serious clinical pathologies, including infections of wounds, colonization of indwelling medical devices including catheters and prosthetic joints, and ventilator-associated pneumonia (VAP) [1–3]. Such infections result in significant morbidity, mortality and healthcare costs meaning that accurate and rapid diagnosis of these infections is critical for the initiation of appropriate treatment [4]. Traditional diagnostic practices for infection rely on bacterial culture, which may take several days to yield a result or techniques such as the polymerase chain reaction (PCR), which have a tendency to be overly sensitive [5, 6].

Developments in optical technologies [7, 8] have opened new avenues for point-of-care diagnosis of disease; however, in the area of bacterial infection, success has been limited by a lack of targeted imaging agents with the required sensitivity, specificity, and compatibility [9, 10]. Gram-positive species such as *Staphylococcus aureus* and *Streptococcus* spp. are responsible for approximately one-third of all VAP in the intensive care unit. Current diagnostic strategies for VAP rely on the interpretation of non-specific clinical symptoms, meaning that half of patients receive an incorrect diagnosis and one-third of cases are missed [3, 11]. Thus, a VAP diagnostic strategy could benefit from technological advances offered by optical endomicroscopy and suitable molecular imaging agents [12, 13].

Here we describe the rational development and characterization of a highly specific, selective, rapidly reporting fluorescent glycopeptide-based imaging agent that has no requirement for a wash or processing step, and demonstrate its utility in detecting Gram-positive bacteria within seconds in a whole explanted human lung. This was achieved using a novel disposable endoscopic delivery/imaging device comprising two delivery capillaries and an optical fiber imaging bundle, allowing the imaging agent to be delivered (< 100 μg) directly into the field-of-view within the alveolar space.

The Gram-positive optical molecular imaging agent was rationally designed based on the clinically utilized glycopeptide antibiotics vancomycin and oritavancin—with the knowledge that oritavancin is produced by reductive amination (with hydrophobic groups) of the natural product chloroeremomycin (an analogue of vancomycin). This gives analogues with far greater activity than chloroeremomycin or vancomycin themselves due to the duality of binding modes—the hydrophobic anchor binding into and disrupting the cell membrane and the classic glycopeptide inhibition of transpeptidation, whilst retaining specificity towards Gram-positive bacteria [14–17].

Thus, our rationale was that the incorporation of an environmentally sensitive hydrophobic dye (merocyanine) by similar chemistry would give highly selective imaging probes (similar in function to oritavancin). Additionally, this chemistry would allow not only selective attachment at a known single site on vancomycin, but it would also leave behind an amino group, thus enhancing solubility and specificity. The merocyanine core was modified with a benzaldehyde type moiety to further mimic the chemistry of chloroeremomycin conversion to oritavancin, which uses an arylaldehyde.

The environmentally sensitive dye utilized was based on merocyanine, which comprises electron donor (D) and acceptor (A) components connected by double bonds (the so-called D-π-A system). This makes the dye very sensitive to changes in polarity and viscosity, while also displaying a high extinction coefficient and quantum yield; thus, it fluoresces brightly in hydrophobic environments (including the bacterial membrane) [18–21].

Merocy-Van is the first example of a red-shifted, environmentally activated bacterial imaging agent, and it demonstrates superior specificity and binding kinetics to Gram-positive bacteria compared with previously reported targeting agents [10, 22]. Moreover, these compounds also had “always-on” fluorophores, precluding them from topical application and rapid bedside imaging. Furthermore, the excitation and emission in the “red” shifts the dye away from the classical green autofluorescence of tissue, making it a promising candidate for imaging applications.

The imaging fiber was fabricated from optical fiber preforms [23] and was subsequently co-packaged with two glass capillaries into a Pebax® outer layer and a polyimide inner layer with a steel braid reinforcement. The distal end was terminated with a stainless steel end cap, and a fiber optic connector at the proximal end for connectivity to the imaging system [24].

In this study, the topical application of Merocy-Van via the capillary channels of our endoscopy device provided a novel delivery approach into ex vivo human lungs and was performed simultaneously with two-color endomicroscopy imaging. The imaging agent delivery and two-color imaging capability enabled both structural and functional information to be collected and exemplifies significant advances in bacterial optical imaging agent design, and in optical endomicroscopy.

## Results

### Synthesis of a Gram-positive specific imaging agent

**Fig. 1:**
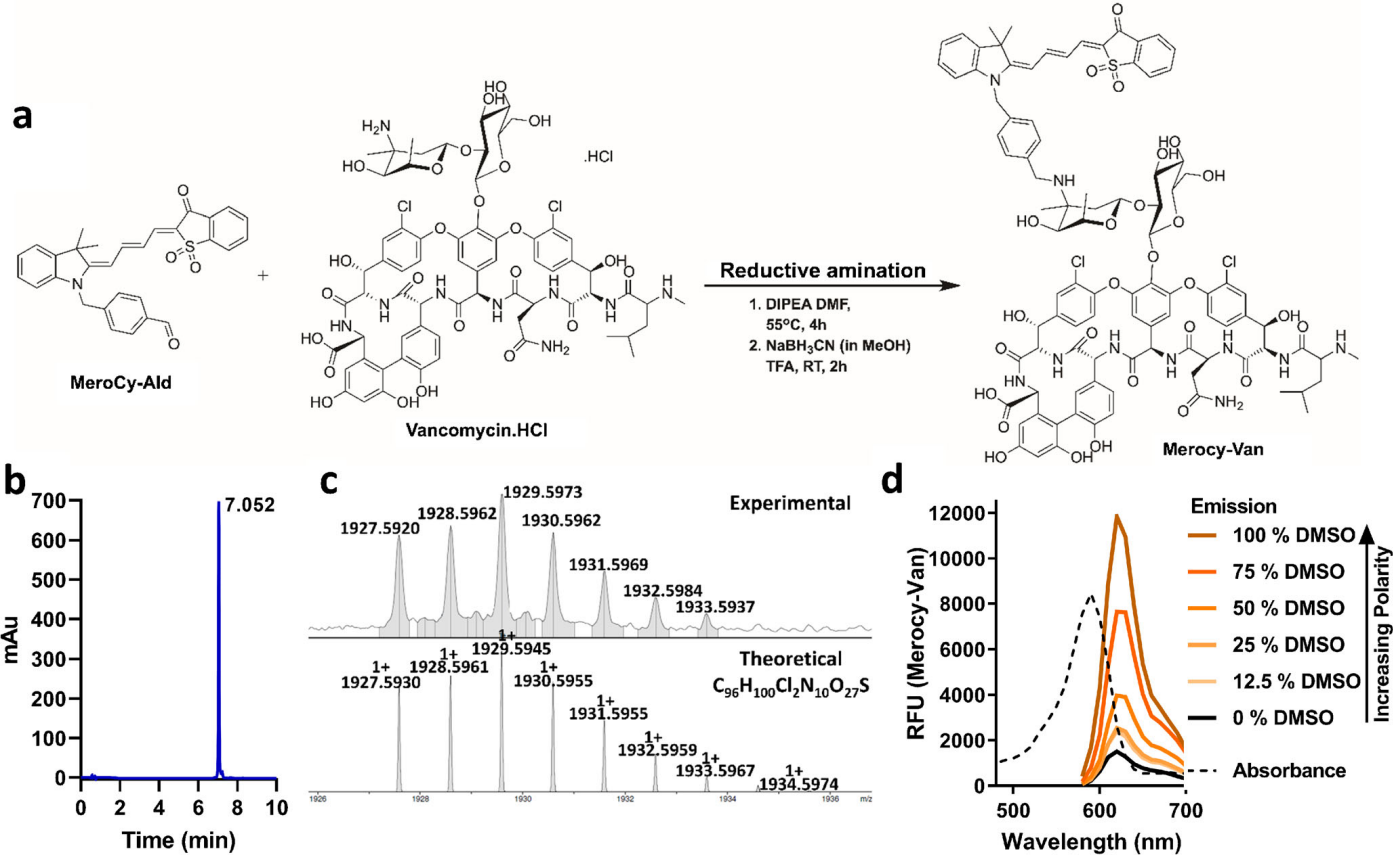
Synthesis and characterization of the environmentally responsive imaging agent. **a** Synthesis of Merocy-Van by reductive amination of vancomycin with the aldehyde dye. **b** HPLC trace of the purified imaging agent (detection at 600 nm). **c** HRMS spectra of Merocy-Van (experimental trace (top) vs theoretical (bottom)). **d** Absorbance (dashed trace) and emission spectra of the imaging agent with increasing levels of DMSO (DMSO/saline, vol/vol)

A benzaldehyde-modified merocyanine was synthesized in three steps (Supporting Information Scheme S1) starting from commercially available 2,3,3-trimethyl-3H-indolenine, which was alkylated with 4-(chloromethyl)benzyl alcohol. Microwave-assisted condensation [20] of the acceptor (indolenine) with the benzothiophene donor gave the benzyl alcohol–modified merocyanine, which was then oxidized (using Dess-Martin periodinane) to give the merocyanine-aldehyde (Merocy-Ald). The imaging agent (Merocy-Van) was generated via reductive amination chemistry [17] of Merocy-Ald via the vancosamine side chain of vancomycin, and purified by reverse phase HPLC (Fig. 1). It proved, as designed, to be environmentally sensitive with an ex_max_ and em_max_ of 590 nm and 620 nm (Fig. 1d).

### Merocy-Van demonstrated selectivity for Gram-positive bacteria

**Fig. 2:**
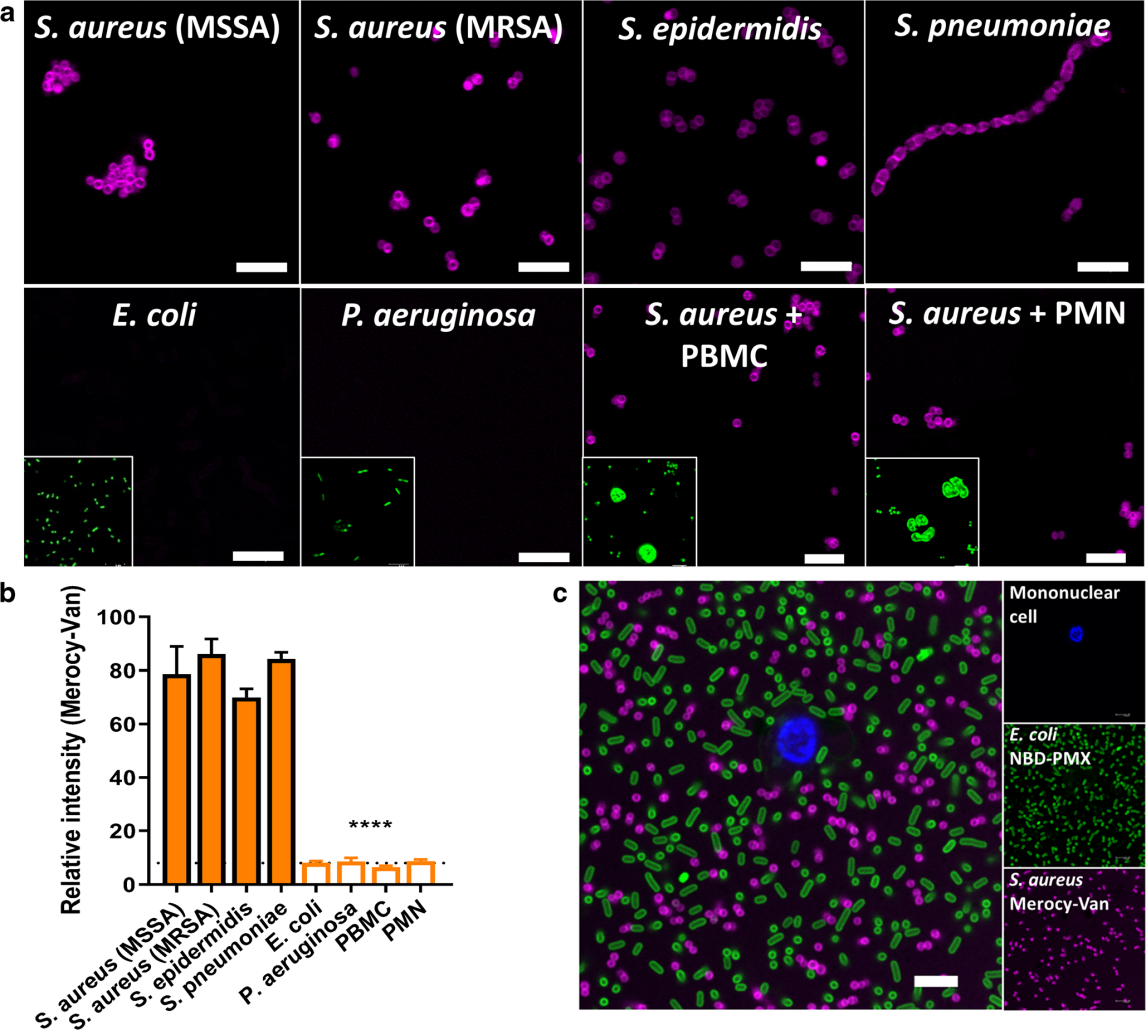
Merocy-Van selectively labels Gram-positive bacteria. **a** *S. aureus*, MRSA, *S. epidermidis*, *S. pneumoniae*, *E. coli*, *P. aeruginosa*, and *S. aureus*/mononuclear cell (PBMC) or granulocyte (PMN) co-cultures were imaged by confocal microscopy following the addition of 5 μM probe (magenta). Image inserts show counterstained bacteria/cells (green). Representative images from nine fields-of-view from three independent repeats. Scale bar = 5 μm. **b** Quantification of images from (**a**) (relative fluorescence intensity from *n* = 90 bacteria, *n* = 20 mononuclear cell, and *n* = 20 granulocyte per condition, one-way ANOVA ****P < 0.0001). Dashed line shows background fluorescence. **c** Wash-free confocal imaging of Gram-negative (green) and Gram-positive (magenta) bacteria with NBD-PMX and Merocy-Van. Primary isolated human mononuclear cells were pre-labeled with Hoechst prior to adding to the confocal chamber. Scale bar = 5 μm, *n* = 3

Selectivity for Gram-positive bacteria was assessed against a panel of clinically relevant Gram-positive and Gram-negative bacteria, and freshly isolated human peripheral blood granulocytes and mononuclear cells. Merocy-Van demonstrated rapid bacterial staining of encapsulated (*S. pneumoniae*) and non-encapsulated Gram-positive bacteria with high signal-to-noise in a concentration-dependent manner (50% max intensity at 0.1 μM) without the need to remove unbound excess probe or requiring a washing procedure (Fig. 2 and Fig. S1). Importantly, Merocy-Van was also able to label MRSA within biofilms of depths of > 10 μM (Fig. S2). Merocy-Van labelling of Gram-positive bacteria was fully orthogonal to the green Gram-negative imaging agent NBD-PMX (PMX (polymyxin)) [13] (Fig. 2c).

### Merocy-Van retained Gram-positive toxicity but was non-cytotoxic

The probe, as expected for oritavancin, caused complete growth inhibition of *S. aureus* and *S. pneumoniae* at 1 μM while the growth rate of *Escherichia coli* remained unaffected (Fig. S3). Merocy-Van was non-toxic and non-hemolytic to eukaryotic cells (Fig. S4), an important pre-requisite for clinical translation.

### Fiber development and performance

**Fig. 3:**
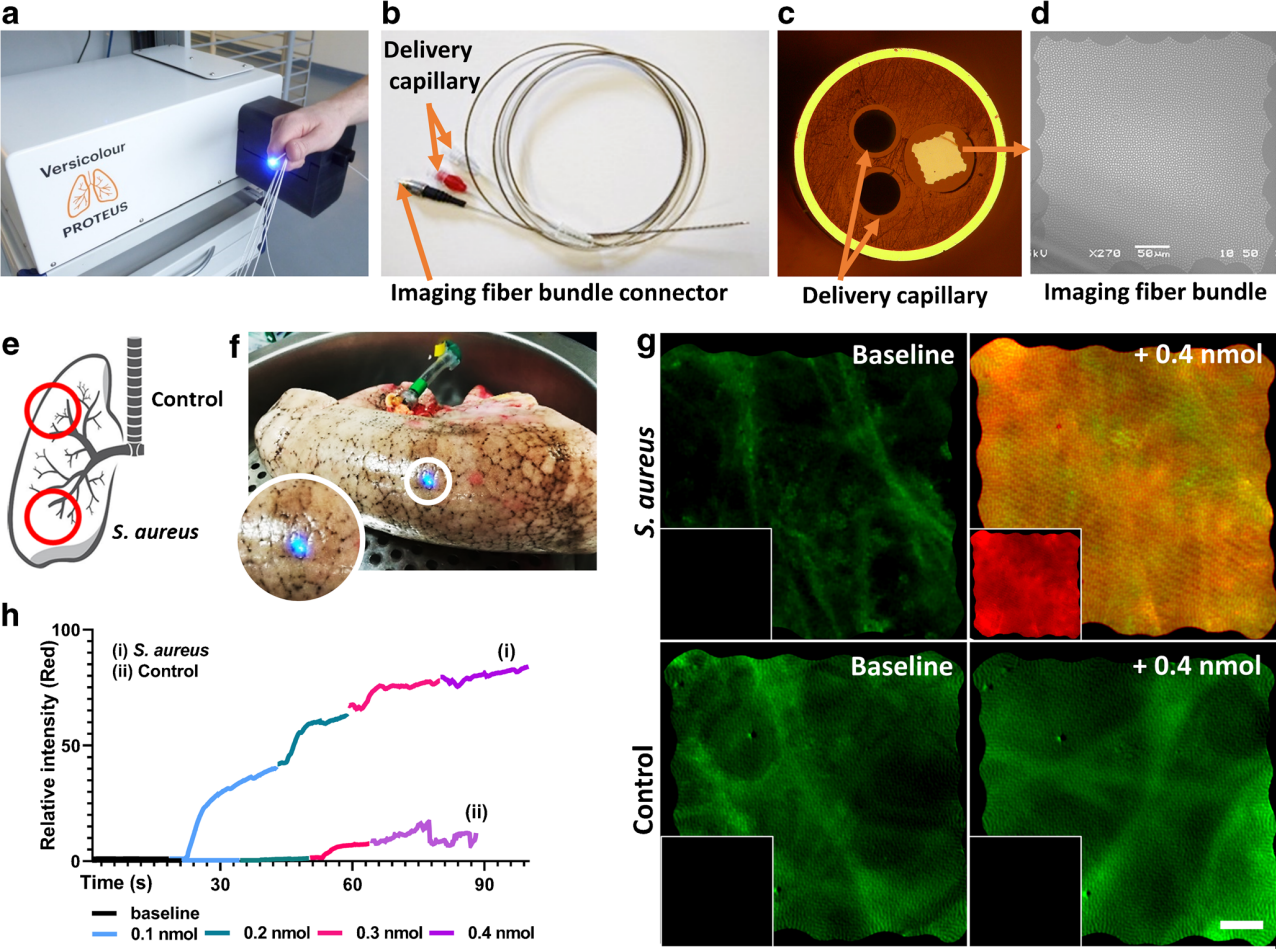
Merocy-Van selectively labels *S. aureus* in an ex vivo human lung model. **a** The endomicroscopy fluorescence imaging system used. **b** The packaged three-in-one fiber-based endomicroscopy device. **c** End of view of the distal tip of the fiber-based endomicroscopy device. The outer diameter of the device is 1.4 mm, and the optical imaging bundle field-of-view is 450 μm (right of the image) and the internal diameter of the glass capillaries is 326 μm. **d** Image of the imaging fiber (8100 cores). **e** Diagram of a human lung and infection/imaging locations used. **f** Image of an ex vivo human lung undergoing ventilation with the imaging device inserted within the distal lung (blue light within the white circle). Insert shows blow-up of device distal end location (blue light). **g** Representative images of real-time Merocy-Van (1 μM) delivery into human ex vivo lung (baseline and following 0.4 nmol delivery shown) within *S. aureus* (top) and control (bottom) lung lobes. Green: capturing lung autofluorescence. Red: capturing activated Merocy-Van. Each large panel shows two-color overlay; insert shows red channel only. Scale bar = 50 μm. **h** Average red fluorescence intensity per frame during real-time delivery of 0.4 nmol Merocy-Van (1 μM) into *S. aureus* and control lung lobes (data shown from lung 1, see Fig. S5 for lungs 2 and 3)

The three-in-one device was fabricated using an imaging fiber (8100 cores with an imaging square diagonal of 450 μm and a center-to-center core separation of 3.5 μm) and two fluid delivery channels formed from a fused silica glass tube drawn down to a 410-μm capillary (inner diameter of 326 μm). The imaging fiber was fabricated from optical fiber preforms produced for the telecommunications industry (Draka/Prysmian OM1 multi-mode preform) and showed improved performance in terms of imaging contrast with no compromise in resolution over conventional imaging fibers formed from significantly more expensive starting glasses [23]. Both the imaging fiber and the capillary fibers were coated, as they were drawn, with a UV curable acrylate polymer to protect the outer surfaces from damage. Biocompatible tubing (Pebax® outer layer and a polyimide inner layer with a steel braid reinforcement) was used to co-package the three individual fibers (2 × capillaries, 1 × imaging fiber), and the three fibers were fixed in place at the distal end inside a 5-mm long stainless steel end cap with an epoxy (Epotek 301). An epoxy fiber splitter was formed at the proximal end (Epotek 301). After the splitter, the three fibers were individually sheathed in the Pebax® tubing; the two fluid channels were terminated with Luer lock fittings and the imaging fiber was terminated with a large bore fiber optic connector (Fig. 3). The delivery system is intended to deliver small boluses to alveolar regions of less than 0.5 cm^3^ for point imaging/sampling. This was achieved, with the fluid channels able to readily deliver 0.5 mL of fluid from a 2-mL syringe in under 20 s in a continuous flow.

### Delivery of Merocy-Van enabled rapid visualization of Gram-positive bacterial in an ex vivo human lung model

Merocy-Van and the optical fiber–based endoscope were evaluated using an explanted ventilated human lung model. *S. aureus* was instilled into the right lower lobe (a common area of the lung for VAP to occur [25]), and *Pseudomonas aeruginosa* was instilled into anatomically distinct lobes as a negative control (Fig. 3e and f). The bacterial inoculum instilled correlated to a bronchoalveolar lavage (BAL) retrieval of ~ 10^4^ colony forming units (CFU) mL^−1^, in line with clinical thresholds for pneumonia diagnosis [12, 26]. Optical endomicroscopy imaging was performed with a wide-field imaging system [24] with appropriate LED and filter sets for the excitation/detection of tissue autofluorescence (green (488 nm, 500–550 nm)) and activated Merocy-Van (red (590 nm, 615–675 nm)) (Fig. 3a). Imaging was simultaneously captured in the two-color channels, with 500 μL of Merocy-Van (1 μM) delivered down the glass capillaries of the optical fiber–based endoscope into the imaging field-of-view. Videos were captured throughout the Merocy-Van instillation process to enable real-time, in situ visualization of probe activation.

In all three individual ex vivo lung experiments, a clear increase red fluorescence was observed for *S. aureus* lung segments following dosing of Merocy-Van (average 20-fold increase within 60 s compared with control lobes) (Fig. 3g and h, and Fig. S5 and S6, and Supporting Information Movies S1, S2, S3). The relative intensity collected from the green channel remained constant throughout. Following the imaging procedure, a BAL was performed and the retrieved sample plated for CFU determination. *S. aureus* was only retrieved from the *S. aureus* lobes, and for each of the three lungs, 10^4^–10^5^ CFU mL^−1^ was recovered.


ESM 2(MP4 5438 kb).


ESM 3(MP4 7132 kb).


ESM 4(MP4 8652 kb).

## Discussion

We have demonstrated the rational design of a red-shifted, environmental, highly selective optical molecular imaging agent to enable real-time detection of Gram-positive bacteria using a dual-color molecular alveoscopy approach. The probe Merocy-Van displayed Gram-positive specificity, no detectable cytotoxicity, and was able to generate high signal-to-noise within seconds of administration into the human distal lung with clinically relevant levels of Gram-positive bacteria. Merocy-Van was dosed directly into the imaging field-of-view without requiring a wash-step, enabling both structural (alveolar location) and functional (Gram-positive bacteria) information to be discerned in real time. Merocy-Van showed superior specificity and binding kinetics to Gram-positive bacteria compared with any other previously reported agents [10, 22]. The choice of fluorophore was critical—many fluorophores carry a surfeit of negative charge or are always “on”—which can inhibit the binding ligand from interacting with fidelity to its target, or generate a high background signal [10]. Our approach to the development of Merocy-Van demonstrates a significant advancement in this area and means that the probe could be utilized in a range of other diagnostic applications.

We evaluated Merocy-Van in a human lung model. The three-in-one optical fiber–based endoscope allows for optical imaging to be proximal to chemical probe delivery as well as providing a capillary for sample removal. Our approach showed it was sensitive and robust, with the alveoscopy procedure performed by three independent clinicians within this study, and offers advantages over conventional lavage processing because the detection and results are presented directly in real time at the site of infection.

Whilst the lung is a large organ and it is not feasible to sample each lobe or sub-section with our fiber-based technique, the clinician can be guided to the bronchopulmonary region of interest based on x-ray or CT scans for fiber-based interrogation and probe delivery, or select the posterior segment of the right lower lobe if changes were widespread, as has previously been demonstrated [13]. The initial clinical application is intended for the bedside evaluation of respiratory deterioration and new pulmonary infiltrates in intubated and ventilated patients in critical care. In these patients, with suspected VAP which has a high morbidity and mortality, antibiotic stewardship is important, yet often broad spectrum antibiotics are prescribed without microbial confirmation of distal lung infection. The intended clinical utility is to provide a high negative predictive value to excluding distal alveolar infection due to Gram-positive bacteria with a threshold to delineate low-grade colonization from de novo infection. The imaging will be performed in a standardized manner, and the technology now requires clinical validation in prospective studies to determine performance and health economic benefit as an adjunct to existing care pathways.

Beyond these initial clinical indications, our approach also allows access to the distal lung with targeted sampling of discrete regions; this has the potential to aid in microbiome research as traditional processing of lavage or tracheal aspirates are not necessarily indicative of the area of interest due to cross-contamination [27, 28]; however, these applications are beyond the scope of this study.

## Conclusion

In conclusion, we have presented a strategy for the in situ detection of Gram-positive bacteria in a whole human ex vivo ventilated lung, paving the way for simultaneous, multiplexed, multicolor imaging in vivo in real time. Combining optical molecular imaging agents and our three-in-one optical fiber–based endoscope constructs could aid point-of-care diagnostic decisions and provide a platform to interrogate dynamic biological processes in situ.

## Methods

### Chemistry and biology reagents and methods

Chemistry and biology reagents and methods are described in detail in the Supporting Information.

### Ethical approval

All experiments using human samples were performed following approval of the appropriate regional ethics committee (REC), NHS Lothian, and the South East Scotland Research Ethics Committee 02 (references 15/HV/013 and 11/SS/0103), and with informed consent. Experiments were conducted within three explanted human lungs (Fig. 3 and Fig. S5 and S6, and Supporting Information Movies S1, S2, S3).

### Optical imaging agent characterization

All imaging agents were solubilized in 0.9% NaCl (used at concentrations of 1 μM or 5 μM). Absorbance and emission spectra were determined on a microplate reader (BioTek Synergy H1 multi-mode reader). Data were collected in duplicate from three independent repeats.

### Human explanted lung model

Human lungs were obtained from solid organ donors after being declined by all UK transplant centers as being unsuitable for transplantation. They were flushed in situ and retrieved as per standard clinical practice for organ retrieval and transported on ice. Lungs were split and single lung ventilation (Drager Savina 300) performed using PEEP 5, tidal volume of 3.5 mL kg^−1^ and a respiratory rate of 12. Following recruitment of the lungs, the bronchoscope was wedged proximally in each lobe, and 2 mL of 10^8^ CFU mL^−1^ *S. aureus* was instilled into the lower lobe, and 2 mL of 10^8^ CFU mL^−1^ *P. aeruginosa* or 2 mL 0.9% NaCl were instilled into the upper or middle lobes as negative controls via a flexible catheter (1.5-mm-diameter APC catheter, Erbe). Following a 60-min incubation, the endoscopic device was passed down the working channel of a bronchoscope to the lobe where saline or bacteria had been instilled, and into the alveolar space. This was confirmed by imaging in the green channel. Five hundred microliters of 1 μM Merocy-Van was instilled into the lung through the capillaries of the endoscopic device within the field-of-view, in 5 × 100 μL (5 × 0.1 nmol) installments. Dual-color videos were captured throughout each instillation of Merocy-Van. Images were captured as previously described [24] with 50-ms exposure and a frame rate of 8 frames per second. The endoscopic device (including internally within the delivery capillaries) was cleaned with 8% H_2_O_2_ between imaging each lobe. Following all imaging, each lobe underwent BAL with 20 mL 0.9% NaCl (Baxter), with a retrieval rate of 40–50%. BAL samples were serially diluted and plated for CFU counting to confirm bacterial presence from each lobe. The study was completed using lungs from three individual donors.

### Human explanted lung model image analysis

Bespoke image analysis tools were developed in-house. To extract fluorescence intensity levels, MATLAB scripts were written that detect and mark the area of the fiber facet as the region of interest (ROI) of an image. The script subsequently removes the background signal (acquired in a calibration step during every experiment) and, in a second step, computes the mean fluorescence intensity levels of the ROI of every image of a given set. Off target frames were removed from analysis.

For display purposes, the image processing we implemented was a method similar to one previously reported [29]. Our implementation first subtracts fiber bundle autofluorescence, then removes high spatial frequency noise from the image, and then corrects for fiber core transmission heterogeneity through normalization. Each video file was brightness- and contrast-enhanced (maintaining the same settings per experiment) and exported to create real-time imaging videos. Individual representative images from each video file were saved as TIFs.

## Electronic supplementary material

ESM 1 (DOCX 10.2 mb).

## Data Availability

Data used within this publication can be accessed at 10.7488/ds/2813.
