# Supplementary material for: Molecular detection of Gram-positive bacteria in the human lung through an optical fiber–based endoscope
Source: Eur J Nucl Med Mol Imaging. 2020 Sep 11;48(3):800–7. doi: 10.1007/s00259-020-05021-4 (PMC7485201; doi:10.1007/s00259-020-05021-4)
Supplement: Supplementary file 4 — (DOCX 10.2 mb). [file 259_2020_5021_MOESM1_ESM.docx]

**Supporting Information**

**Molecular Detection of Gram-positive Bacteria in the Human Lung through an Optical Fiber Based Endoscope**

Bethany Mills^1^, Alicia Megia-Fernandez^2^, Dominic Norberg^1^, Sheelagh Duncan^1^, Adam Marshall^1^, Ahsan R. Akram^1^, Thomas Quinn^1^, Irene Young^1^, Annya M. Bruce^1^, Emma Scholefield^1^, Gareth O. S. Williams^1^, Nikola Krstajić^1^, Tushar R. Choudhary^1,3^, Helen E. Parker^1,4^, Michael G. Tanner^1,5^, Kerrianne Harrington^6^, Harry A.C. Wood^6^, Timothy A. Birks^6^, Jonathan C. Knight^6^, Christopher Haslett^1^, Kevin Dhaliwal^1^, Mark Bradley^2^*, Muhammed Ucuncu^2,7^*, and James M. Stone^6^*

^1^ Centre of Inflammation Research, Queen's Medical Research Institute, University of Edinburgh, 47 Little France Crescent, Edinburgh EH16 4TJ, UK.

^2^ School of Chemistry, University of Edinburgh, Joseph Black Building, David Brewster Road, Edinburgh EH9 3FJ, UK.

^3^ The Roslin Institute and Royal (Dick) School of Veterinary Studies, University of Edinburgh, Edinburgh, UK.

*^4^* Department of Applied Physics, Royal Institute of Technology, KTH, 10691 Stockholm, Sweden.

^5^ Scottish Universities Physics Alliance (SUPA), Institute of Photonics and Quantum Sciences, Heriot-Watt University, Edinburgh EH14 4AS, UK.

^6^ Centre for Photonics and Photonic Materials, Department of Physics, University of Bath, BA2 7AY, Bath, UK.

^7^ Department of Analytical Chemistry, Faculty of Pharmacy, Izmir Katip Celebi University, Izmir, Turkey.

*Corresponding authors: [Mark.Bradley@ed.ac.uk](mailto:Mark.Bradley@ed.ac.uk), [beth.mills@ed.ac.uk](mailto:beth.mills@ed.ac.uk), [muhammed.ucuncu@ikcu.edu.tr](mailto:muhammed.ucuncu@ikcu.edu.tr) and [J.M.Stone@bath.ac.uk](mailto:J.M.Stone@bath.ac.uk)

**Table of Content**

1. Experimental Procedures
   1. Chemistry
      1. Materials and Methods
      2. Synthesis
   2. **Biology**
      1. Ethical Approval
      2. Biological Assay Reagents
      3. Peripheral Blood Cell Isolation
      4. Cellular Toxicity
      5. Bacterial Culture
      6. Confocal Laser Scanning Microscopy Assessment of Imaging Agent Binding
      7. Bacterial Biofilm
      8. Bacterial Killing
      9. Data plotting and statistical analysis
2. **Supplementary Schemes and Figures**

Scheme S1. Synthetic route to Merocy-Van

Fig. S1. Merocy-Van labels *S. aureus* in a concentration dependent manner

Fig. S2. Merocy-Van labels MRSA biofilms *in vitro*.

Fig. S3. Merocy-Van demonstrates enhanced toxicity towards Gram-positive bacteria compared to vancomycin

Fig. S4. Merocy-Van is non-toxic or hemolytic to eukaryotic cells

Fig. S5. Merocy-Van selectively labelled *S. aureus* in an *ex vivo* human lung model 1.

Fig. S6. Merocy-Van selectively labelled *S. aureus* in an *ex vivo* human lung model 2 and lung 3.

1. **Supplementary Characterization Data**
   1. HPLC & HRMS
   2. ^1^H & ^13^C NMR
2. **Movies**
   1. Movie S1: *In situ* optical molecular imaging of control lung lobe and *S. aureus* instilled lung lobe during instillation of Merocy-Van, Lung 1.
   2. Movie S2: *In situ* optical molecular imaging of control lung lobe and *S. aureus* instilled lung lobe during instillation of Merocy-Van, Lung 2.
   3. Movie S3: *In situ* optical molecular imaging of control lung lobe and *S. aureus* instilled lung lobe during instillation of Merocy-Van, Lung 3.
3. **References**
4. Experimental Procedures
   1. Chemistry
      1. Materials and Methods

Chemicals used in this work were purchased from Sigma Aldrich, Merck, Acros, VWR, GL Biochem, Gibco and Fisher Scientific. Commercially available reagents were used without further purification. NMR spectra were recorded at 298 K in deuterated solvents using Bruker AVA500 spectrometer operating at 500 MHz for ^1^H and 126 MHz for ^13^C. Chemical shifts are reported in ppm and are referenced to residual non-deuterated solvent. Normal phase column chromatography was carried out on silica gel 60 (230–400 mesh). Analytical reverse-phase high-performance liquid chromatography (RP–HPLC) was performed on an Agilent 1100 system equipped with a Phenomenex Kinetex® 5 µm XB-C18 100 Å LC Column (50 × 4.6 mm) with a flow rate of 1 mL/min. Method 1: A gradient of H_2_O/CH_3_CN (95/5) to H_2_O/CH_3_CN (5/95) with 0.1% HCOOH, over 6 min, holding at 95% CH_3_CN for 3 min, followed by 1 min isocratic elution. Method 2: A gradient of H_2_O/CH_3_CN (95/5) to H_2_O/CH_3_CN (20/80) with 0.1% CF_3_COOH, over 10 min, then to H_2_O/CH_3_CN (5/95), over 4 min, followed by 1 min isocratic elution with detection at 600 nm and by evaporative light scattering. Preparative RP–HPLC was performed on an Agilent 1100 system equipped with a Phenomenex Kinetex® 5µm XB-C18 reverse-phase column (150 × 21.2 mm, 5 μm) with a flow rate of 10 mL/min. A gradient of H_2_O/CH_3_CN (80/20) to H_2_O/CH_3_CN (50/50) with 0.1% CF_3_COOH, over 15 min then from H_2_O/CH_3_CN (50/50) to H_2_O/CH_3_CN (5/95) over 4 min, followed by 1 min isocratic elution with detection at 600 nm. Electrospray ionization mass spectrometry (ESI–MS) analyses were carried out on an Agilent Technologies LC/MSD Series 1100 quadrupole mass spectrometer (QMS) in an ESI mode. High Resolution MS were performed on a Bruker microTOF focus II mass spectrometer.

- - 1. **Synthesis**

***Compound 1:*** To a solution of potassium iodide (277 mg, 1.6 mmol) in acetonitrile (1.6 mL) 4-(chloromethyl)benzyl alcohol (171.6 mg, 1.1 mmol) was added and reaction stirred at 50^o^C for 1 hour. 2,3,3-Trimethyl-3*H*-indole (159.2 mg, 1 mmol) was added to this solution and stirred for a further 45 hour at reflux. Then, the solvent was evaporated and filtered over a pad of silica washing with 200 mL of hexane–ethylacetate (4:1) then 500 mL of dichloromethane–methanol (10:1). The dichloromethane-methanol fraction was evaporated, dissolved in small amount of chloroform and precipitated by hexane (3x). The structure of product was confirmed by ^1^H, ^13^C NMR and mass spectrometry from crude and used without further purification.

**^1^H NMR (500 MHz, [D_6_]-DMSO) δ ppm.** 7.86 (d, *J* = 7.5 Hz, 1H), 7.80 (d, *J* = 7.8 Hz, 1H), 7.60 (t, *J* = 7.3 Hz, 1H), 7.55 (t, *J* = 7.4 Hz, 1H), 7.36 (q, *J* = 8.2 Hz, 4H), 5.79 (s, 2H), 4.48 (s, 2H), 2.97 (s, 3H), 1.59 (s, 6H). **^13^C NMR (126 MHz, [D_6_]-DMSO) δ ppm.** 197.9, 143.2, 141.9, 141.0, 130.3, 129.5, 128.9, 127.3, 127.2, 123.6, 115.9, 62.3, 54.4, 50.51, 22.1, 14.4. **MS** (ES)^+^ *m/z* 281.2 [M+H]^+^ **HRMS** (ESI)^+^ calcd for C_19_H_22_NO [M]^+^ *m/z* 280.1696 found *m/z* 280.1688. **HPLC** t_R_*_(analytical)_* 2.46 min. (Method 1)

***Compound 2:*** Benzo[*b*]thiophen-3(2*H*)-one 1,1-dioxide (364 mg, 2.0 mmol, 1.0 equiv.) together with 1,1,3,3-tetramethoxypropane (1.65 mL, 10.0 mmol, 5.0 equiv.) were added to a vial followed by TFA (15 μL, 0.2 mmol, 0.1 equiv.). The vial was sealed and heated in the MW (150°C, 15 min). The reaction was allowed to cool to room temperature and the precipitate was filtered and washed with cold hexane/ether 3:1 to give (*E*)-2-((*E*)-3-methoxyallylidene)benzo[*b*]thiophen-3(2*H*)-one 1,1-dioxide (255 mg, 51%) as a red solid. ^1^H-NMR spectra matched those previously reported.^1^

***Merocy-Alc:*** Compound 1 (250 mg, 0.89 mmol) and Compound 2 (171 mg, 0.68 mmol) were mixed in microwave vial with NaOAc (56 mg, 0.68 mmol) in MeOH–DCM (4 mL, (1:1, v/v)). The vial was capped and heated to 75^o^C for 30 min. The reaction mixture cooled to room temperature and then the solvent evaporated under reduced pressure. The crude compound was purified by column chromatography (SiO_2_/100:1 Dichloromethane–Methanol). (Purple solid, 186 mg, 55%)

**^1^H NMR (500 MHz, [D_6_]-DMSO) δ ppm** 8.24 (t, *J* = 13.0 Hz, 1H), 8.03 – 7.99 (m, 2H), 7.96 – 7.79 (m, 3H), 7.56 (dd, *J* = 7.5, 1.2 Hz, 1H), 7.35 – 7.26 (m, 3H), 7.22 – 7.20 (m, 3H), 7.15 (t, *J* = 7.4 Hz, 1H), 6.57 (t, *J* = 13.0 Hz, 1H), 6.32 (d, *J* = 13.3 Hz, 1H), 5.31 (s, 2H), 4.45 (s, 2H), 1.69 (s, 6H). **^13^C NMR (126 MHz, [D_6_]-DMSO) δ ppm.** 175.9, 171.2, 155.0, 143.9, 143.1, 142.6, 142.0, 140.5, 134.9, 133.9, 133.8, 133.0, 128.2, 127.0, 126.2, 123.8, 123.2, 122.3, 120.4, 118.2, 116.1, 110.3, 101.1, 62.6, 48.3, 45.7, 27.4. **MS** (ES)^+^ *m/z* 498.1 [M+H]^+^ **HRMS** (ESI)^+^ calcd for C_30_H_28_NO_4_S [M+H]^+^ *m/z* 498.1734 found *m/z* 498.1717. **HPLC** t_R_*_(analytical)_* 5.30 min. (Method 1)

***Merocy-Ald:*** To a solution of Merocy-Alc (149.1 mg, 0.3 mmol) in 25 mL of dry DCM Dess-Martin periodinane (152.4 mg, 0.36 mmol) was added and the reaction mixture stirred at room temperature under N_2_ atmosphere. Progress of the reaction was monitored by analytical TLC. After complete conversion the reaction mixture was quenched with 20% aqueous Na_2_S_2_O_3_ (6 mL) and saturated NaHCO_3_ (24 mL). The mixture was stirred for 30 min then, extracted with DCM (3 x 150 mL). The combined organic layers were dried over MgSO_4_ and concentrated under reduced pressure. Title product purified by column chromatography (SiO_2_/100:1 Dichloromethane–Methanol). (Purple solid, 134 mg, 90 %).

**^1^H NMR (500 MHz, [D]-Chloroform) δ ppm.** 10.01 (s, 1H), 8.00 (d, *J* = 7.4 Hz, 1H), 7.95 – 7.86 (m, 3H), 7.82 – 7.64 (m, 4H), 7.34 (t, *J* = 7.2 Hz, 3H), 7.29 – 7.21 (m, 1H), 7.13 (td, *J* = 7.5, 0.9 Hz, 1H), 6.82 (d, *J* = 7.9 Hz, 1H), 6.75 (t, *J* = 13.1 Hz, 1H), 5.80 (d, *J* = 12.9 Hz, 1H), 5.07 (s, 2H), 1.72 (s, 6H). **^13^C NMR (126 MHz, [D]-Chloroform) δ ppm.** 191.5, 178.0, 168.1, 152.0, 144.2, 143.9, 143.1, 141.3, 139.8, 136.4, 134.9, 133.8, 133.6, 130.9, 128.7, 126.9, 124.1, 123.7, 122.4 (x2), 120.9, 117.7, 108.7, 100.0, 48.1, 46.7, 28.7. **MS** (ES)^+^ *m/z* 496.2 [M+H]^+^ **HRMS** (ESI)^+^ calcd for C_30_H_26_NO_4_S [M+H]^+^ *m/z* 496.1577 found *m/z* 496.1565. **HPLC** t_R_*_(analytical)_* 5.83 min. (Method 1)

***Merocy-Van:*** Reductive amination reaction was carried out using literature method.^2^ To a solution of Merocy-Ald (148.9 mg, 0.3 mmol) in DMF (8.8 mL) Vancomycin.HCl (217.4 mg 0.15 mmol) and DIPEA (66 µL) were added and the reaction mixture stirred for 4 hours at 55^o^C. Then, the reaction mixture was cooled to room temperature and NaBH_3_CN (17.6 mg, 0.28 mmol) in MeOH (1.76 mL) and TFA (66 µL) were added and the reaction mixture was allowed to stir at room temperature for further 2 hours. The solvent was removed under reduced pressure and solid washed with diethyl ether (3 x 15 mL). The crude compound purified by preparative RP-HPLC (C18/Water–Acetonitrile (0.1% TFA)). (Purple solid, 84 mg, 29%). **HRMS** (ESI)^+^ calcd for C_96_H_101_Cl_2_N_10_O_27_S [M+H]^+^ *m/z* 1929.5945 found *m/z*1929.5973. **HPLC** t_R_*_(Prep)_* 13.49 min. t_R_*_(analytical)_* 7.05 min (Method 2)

- 1. **Biology**
     1. **Ethical Approval**

All experiments using human samples *in vitro* and *ex vivo* were performed following approval of the appropriate regional ethics committee (REC), NHS Lothian and the South East Scotland Research Ethics Committee 02 (references 15/HV/013 and 11/SS/0103), and with informed consent.

- - 1. **Biological Assay Reagents**

Unless otherwise stated, all biological assay reagents and kits were purchased from Merck.

- - 1. **Peripheral Blood Cell Isolation**

Platelet rich plasma (PRP), erythrocytes, granulocytes and mononuclear cells were isolated from the blood of healthy human volunteers as previously described^3^. The number of retrieved granulocytes and mononuclear cells was determined with NucleoCounter NC-1000 (Chemo Metec). Cells were resuspended at a concentration of 20 x 10^6^ mL^-1^ in 0.9 % NaCl with 0.9 mM CaCl_2_. Where appropriate, cells were counterstained with SYTO9, or Hoechst (all ThermoFisher Scientific) as appropriate, according to manufacturer’s instructions. Cells were seeded into confocal imaging chambers (IBIDI, 80821) coated with fibronectin at 50,000 cells per well. All experiments were carried-out using three independent donors (one donor per independent repeat).

- - 1. **Cellular Toxicity**

Toxicity of Merocy-Van was determined by hemolysis assay and a WST-1 assay. Merocy-Van was incubated with 0.7 % primary isolated human erythrocytes for 60 min at 37 °C. 0.7 % erythrocytes were serially diluted in 0.9 % NaCl and sonicated to produce a lysis standard curve. Supernatants were harvested by centrifugation, 400 x *g,* 5 min. Lysis was determined spectroscopically at 450 nm using the microplate reader. For the WST-1 assay, A549 cells were seeded into a 24-well plate at 4 x 10^4^ cells mL^-1^ in complete DMEM. Cells were incubated at 37 °C, 5 % CO_2_ for 24h. Merocy-Van was added for 2h. No-probe and 70% MeOH acted as controls. Cells were washed and incubated with WST-1 reagent, 37 °C, 30 min. Supernatants were removed and measured at 450 nm by microplate reader. All experiments were performed in duplicate or triplicate and repeated independently thrice.

- - 1. **Bacterial Culture**

*Staphylococcus aureus* ATCC 25923 (MSSA)*,* Methicillin Resistant *Staphylococcus aureus* ATCC252 (MRSA)*, Staphylococcus epidermidis* (clinical isolate), *Streptococcus pneumoniae* J3062 (clinical isolate), *Escherichia coli* ATCC 25922 and *Pseudomonas aeruginosa* 3284 (clinical isolate from a VAP patient) were sourced from an in-house strain collection. Identification of clinical bacterial species was confirmed through the bacterial diagnostic laboratories, Royal Infirmary of Edinburgh, Edinburgh, United Kingdom. *S. pneumoniae* were grown directly from colony in Tryptic Soy Broth (TSB) (Oxoid) TSB, 37 °C, 5 % CO_2_ with shaking. All other bacteria were grown overnight in Luria broth (LB) (Oxoid) from a single colony, 37 °C with shaking. Cultures were diluted 1:100 and grown to mid-log phase. OD_595_ 1 was harvested by centrifugation and washed in 0.9 % NaCl (Baxter).

- - 1. **Confocal Laser Scanning Microscopy Assessment of Imaging Agent Binding**

Bacteria were grown and harvested as outlined above. Bacteria were counterstained with 5 µM SYTO9 (ThermoFisher Scientific) as appropriate and added to confocal imaging chambers coated with poly-d-lysine with 5 µM Merocy-Van. Human cells were prepared as described above and added as required and imaged by confocal laser scanning microscopy (CLSM) (Leica SP8) without a wash-step. CLSM was performed under oil emersion 63x objective (HC PL APO CS2 63x 1.40 oil). Hoechst was excited at 405 nm (detected 410-480 nm), NBD and Syto9 were excited at 488 nm (detected 500-560 nm) and merocyanine was excited at 561 nm (detected 570-660 nm). Detection was performed with HyD detectors. Images were processed using LAS X (Leica). Maximum fluorescence amplitude was determined for a 10 bacteria per frame, 3 frames per experiment. All data was collected independently thrice.

- - 1. **Bacterial Biofilm**

*S. aureus* MRSA was grown overnight in Brain Heart Infusion (BHI) under standard conditions. Overnight cultures were diluted to OD_595_ 0.01 in BHI supplemented with 10 % PRP. Biofilms were cultivated in static confocal imaging chambers (IBIDI, 80821) for 48 h, 37 °C, 200 µL per well. Biofilms were rinsed in saline and labelled with Syto9 (5 µM) and Merocy-Van (5 µM) and imaged immediately by CLSM as outlined above. 3D renders were performed with IMARIS software (Oxford Instruments). Data was collected independently thrice.

- - 1. **Bacterial Killing**

Bacterial cultures were diluted to OD_595_ 0.001 in Muller Hinton (MH) broth in a 96-well plate. Merocy-Van or vancomycin were added as appropriate and bacterial growth was measured using the microplate reader by absorbance at OD_600_ 37 °C with shaking for 18 h. Conditions were plated in duplicate and the experiments were repeated thrice.

- - 1. **Data Plotting and Statistical Analysis**

Statistical analyses were performed using Prism 8 (GraphPad Software Inc., La Jolla, CA, USA). Where appropriate, analyses were performed using one-way ANOVA. Unless otherwise stated error bars show standard error of the mean (s.e.m).

1. **Supplementary Schemes and Figures**


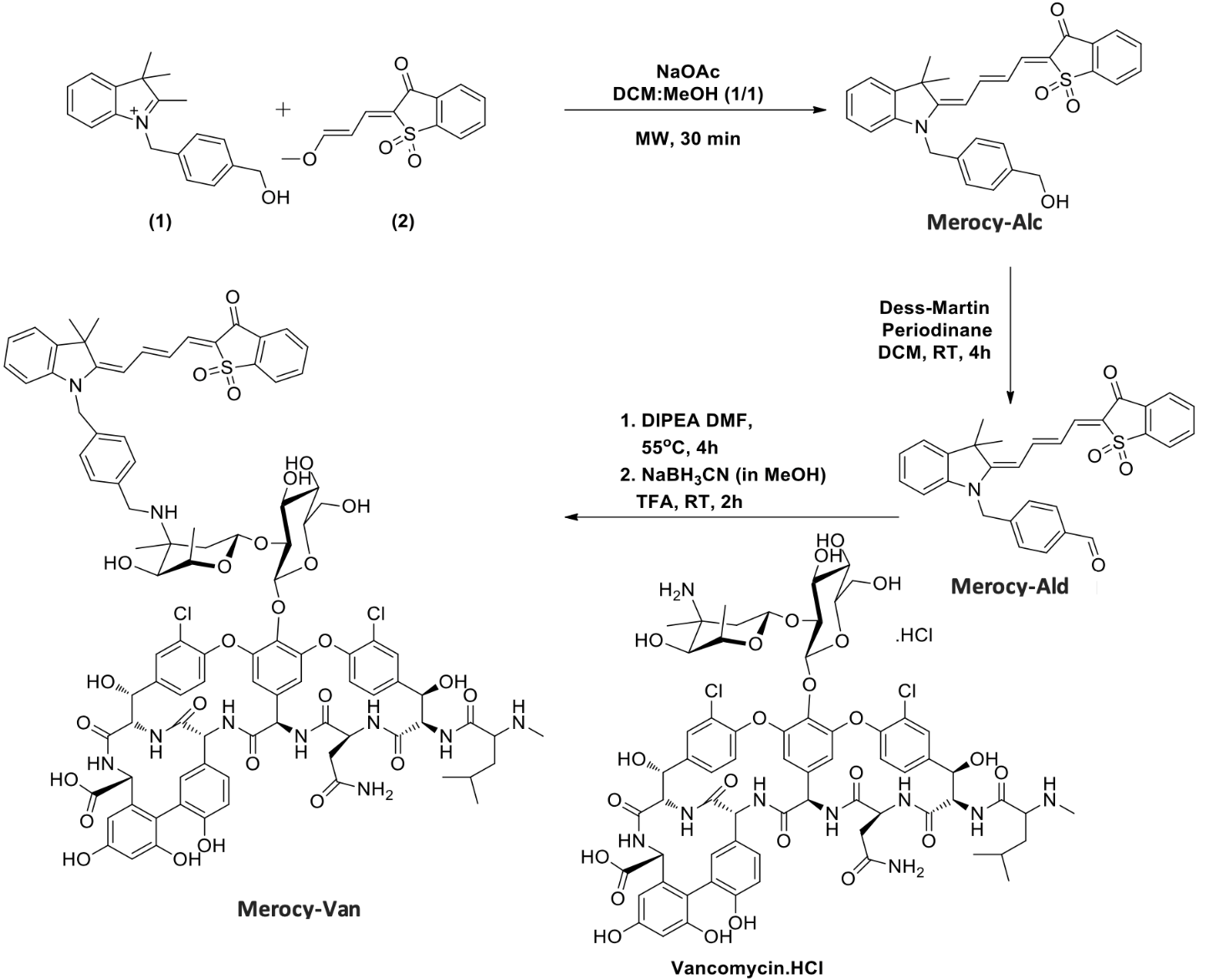


**Scheme S1.** Synthetic route to **Merocy-Van.**


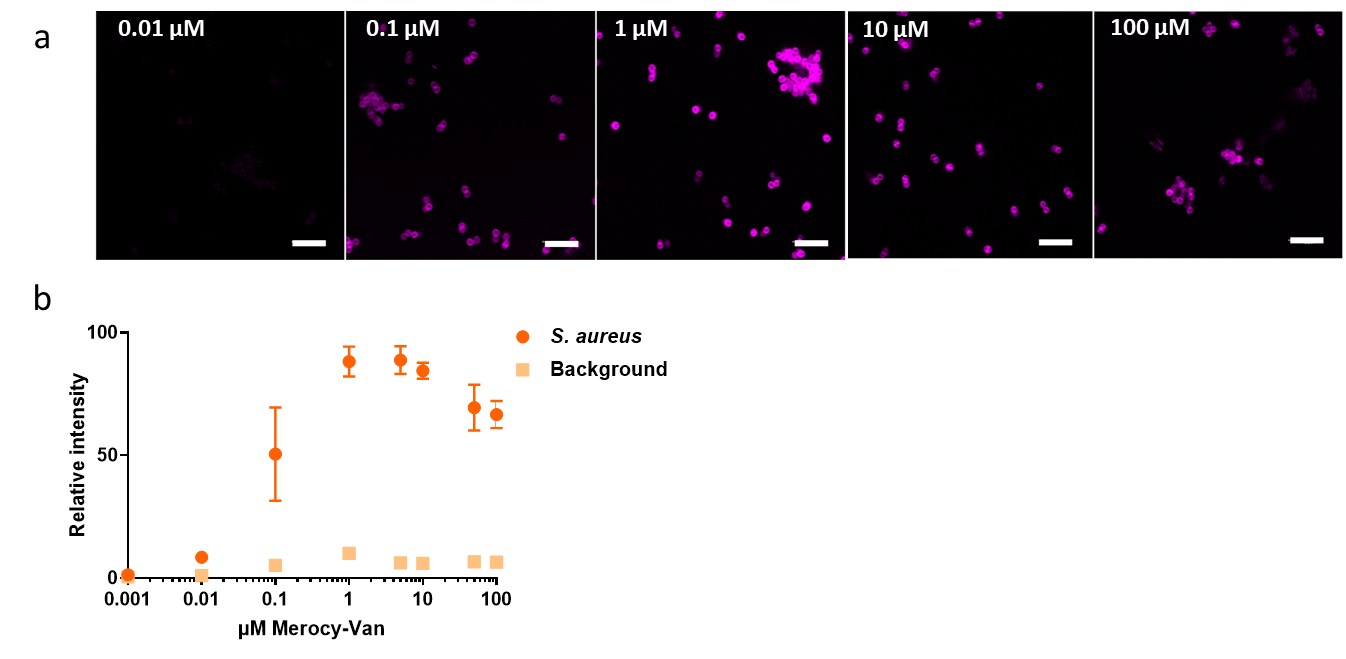


**Figure S1.** **Merocy-Van labels *S. aureus* in a concentration dependent manner. (a)** Representative fluorescence imaging by benchtop confocal imaging of *S. aureus* with increasing concentrations of Merocy-Van (magenta). Scale bar = 5 µm. **(b)** Quantification of fluorescence intensity from (a) with quantification performed on 3 fields-of-view per experiment, examining both bacterial intensity and background intensity, experiments were repeated independently thrice (n=90 bacteria, n=9 background). Data shows mean and s.e.m.


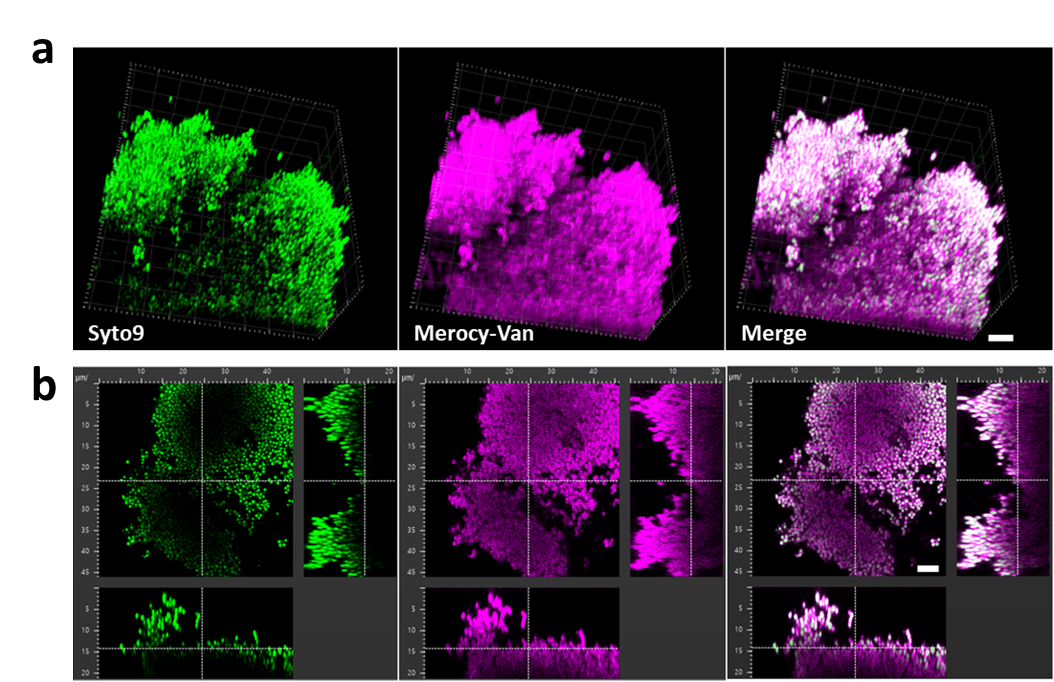


**Figure S2. Merocy-Van labels MRSA biofilms in vitro**. 48 h MRSA biofilms were counterstained by Syto9 (5 µM). Biofilms were imaged by confocal microscopy following addition of Merocy-Van (5 µM). **a** 3D render and **b** orthogonal view of a representative z-stack is shown. Scale bar = 5 µm, n=3


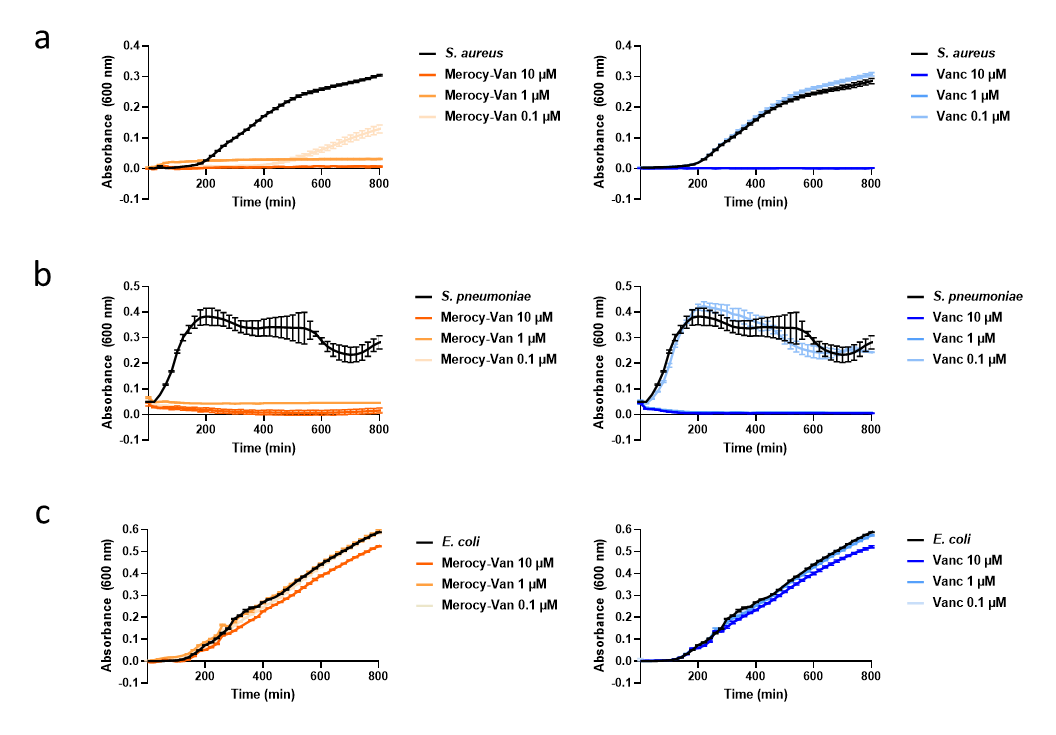


**Figure S3.** **Merocy-Van demonstrates enhanced toxicity towards Gram-positive bacteria compared to vancomycin.** Representative growth kinetics of **(a)** *S. aureus*, **(b)** *S. pneumoniae* and **(c)** *E. coli* in the presence of increasing concentrations of Merocy-Van (orange) or vancomycin (blue). Growth was determined by absorbance at 600 nm. All assays were performed in triplicate and repeated thrice. Bars show mean and s.e.m.


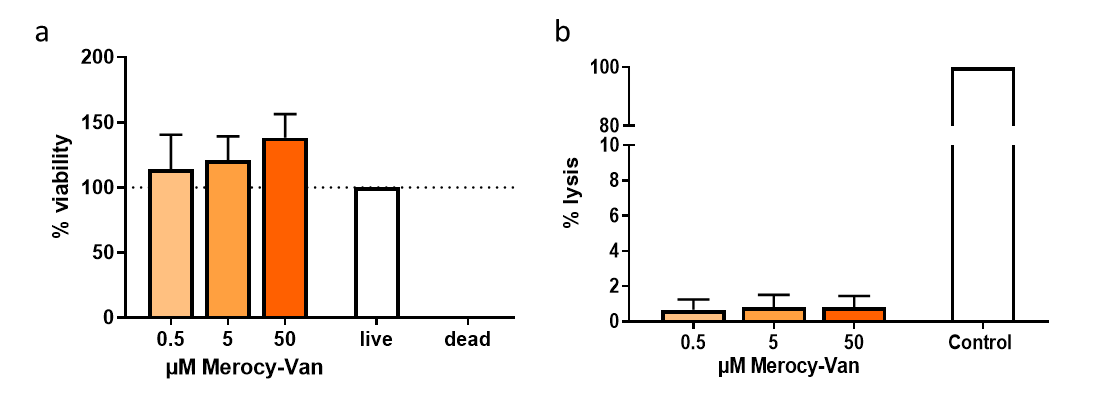


**Figure S4.** **Merocy-Van is non-toxic or hemolytic to eukaryotic cells. (a)** A549 cells were challenged with increasing concentrations of Merocy-Van (orange bars). Viability was assessed by WST-1 assay. **(b)** Hemolytic activity Merocy-Van was determined using freshly isolated human erythrocytes incubated with increasing concentrations of Merocy-Van (orange bars). All assays were performed in triplicate and repeated thrice. Bars show mean and s.e.m of combined data.


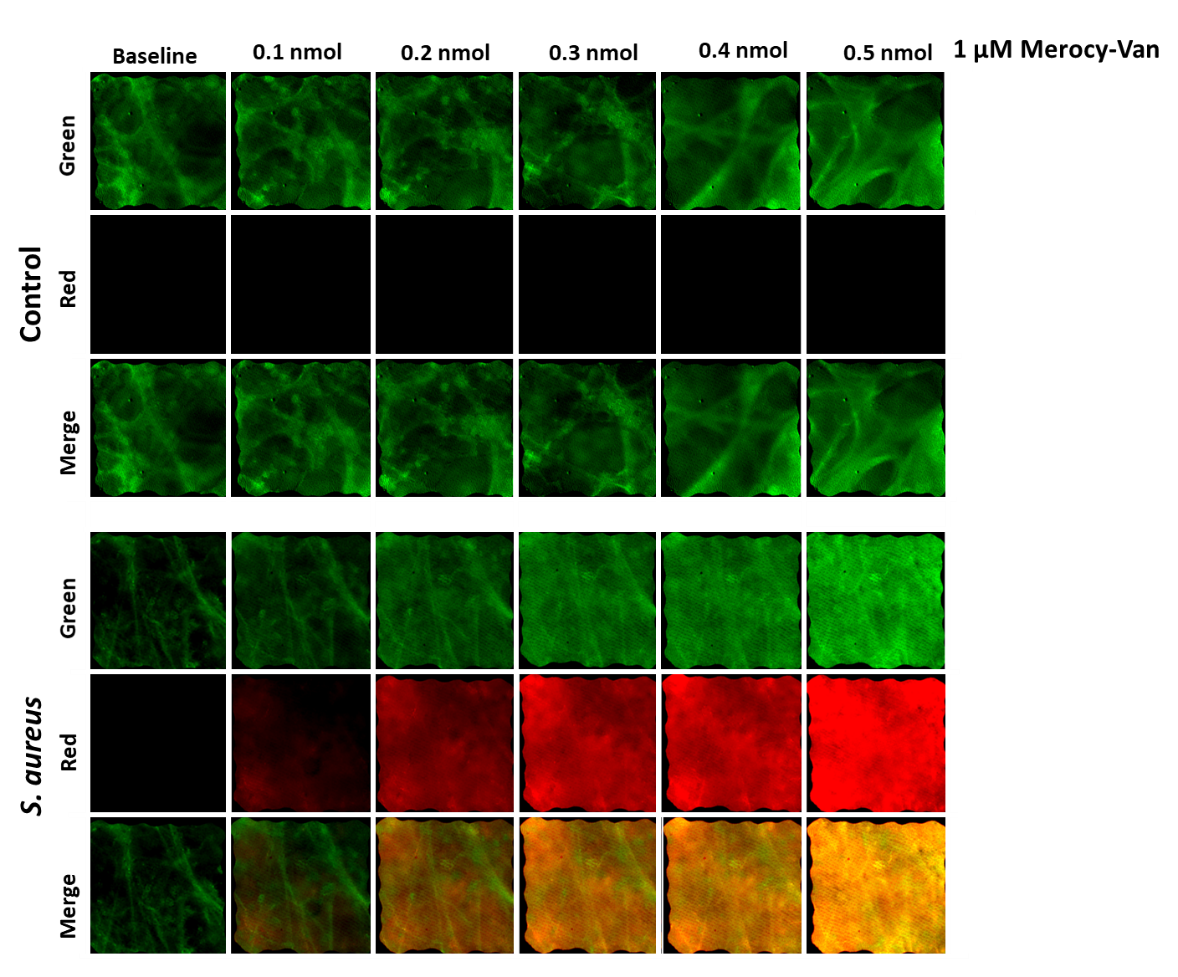


**Figure S5. Merocy-Van selectively labelled *S. aureus* in an *ex vivo* human lung model.** Representative images and quantification of real-time Merocy-Van delivery (1 µM in Saline, 0.1 nmol to 0.5 nmol) into human *ex vivo* whole lung model. Lung 1.


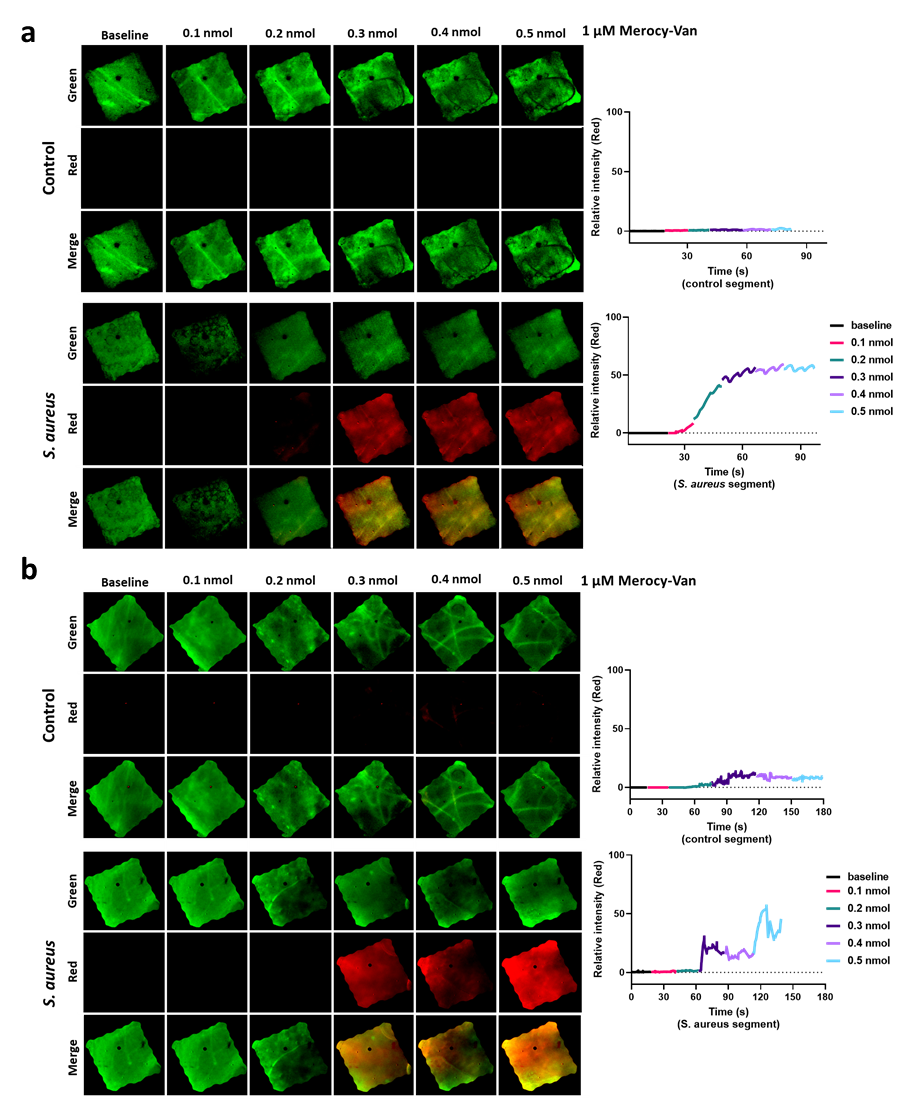


**Figure S6. Merocy-Van selectively labelled *S. aureus* in an *ex vivo* human lung model.** Representative images and quantification of real-time Merocy-Van delivery (1 µM in Saline, 0.1 nmol to 0.5 nmol) into human *ex vivo* whole lung model. **(a)** Lung 2 **(b)** Lung 3.

1. **Supplementary Characterization data**
   1. **HPLC & HRMS data**


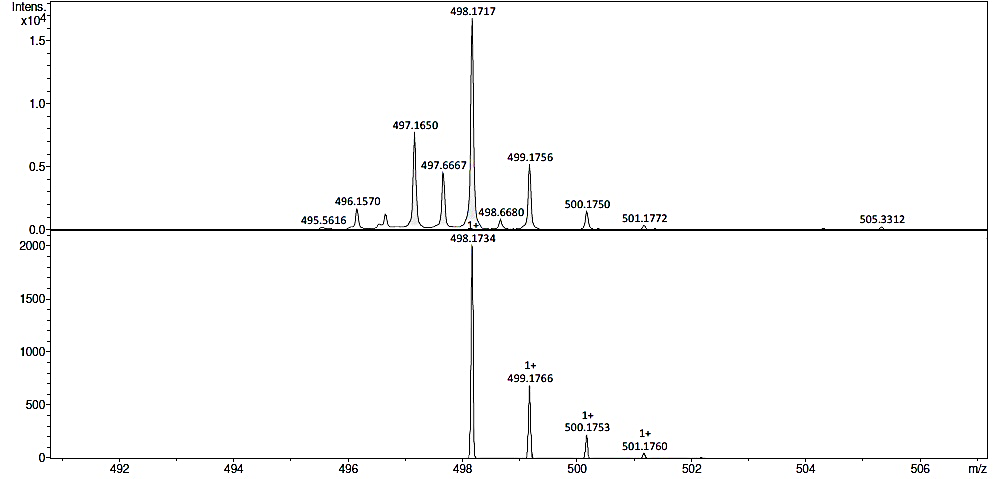


**Experimental**

**Theoretical**

**C_30_H_28_NO_4_S**

**a**

**b**

**Figure S6. Merocy-Alc characterization. (a)** RP-HPLC Analysis of Merocy-Alc (detection at 600 nm) and **(b)** HRMS traces of Merocy-Alc (The inset: experimental (top) vs theoretical (bottom)).


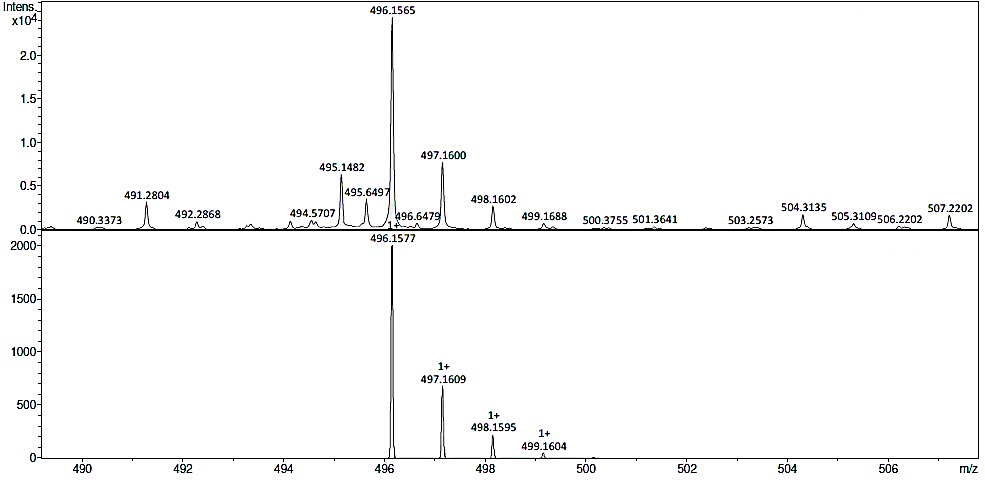


**Theoretical**

**C_30_H_26_NO_4_S**

**Experimental**

**a**

**b**

**Figure S7. Merocy-Ald characterization. (a)** RP-HPLC Analysis of Merocy-Ald (detection at 600 nm) and **(b)** HRMS traces of Merocy-Ald (The inset: experimental (top) vs theoretical (bottom)).


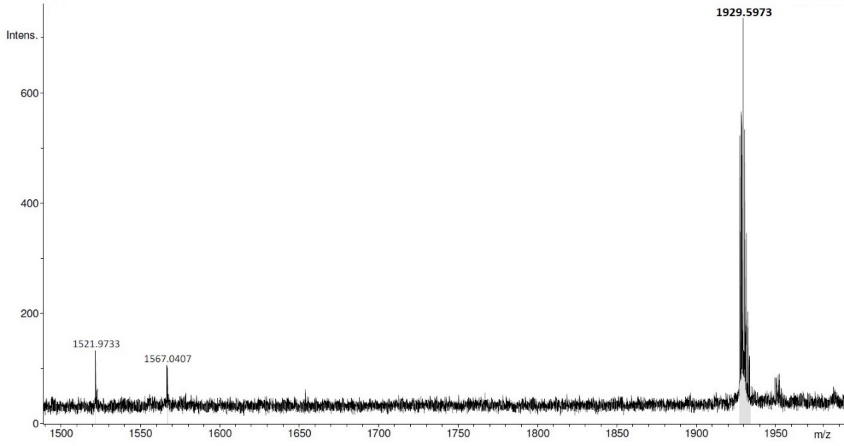

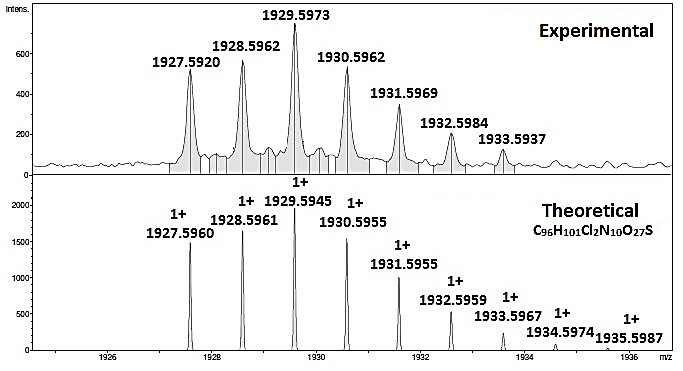


**a**

**b**

**Figure S8. Merocy-Van characterization. (a)** RP-HPLC Analysis of Merocy-Van (detection at 600 nm) and **(b)** HRMS traces of Merocy-Van (The inset: experimental (top) vs theoretical (bottom)).

- 1. **^1^H & ^13^C NMR**

**a**

**b**

**Figure S9. Compound 1 characterization. (a)** ^1^H and **(b)** ^13^C NMR spectra in DMSO-d_6_.

**a**

**b**

**Figure S10. Merocy-Alc characterization. (a)** ^1^H and **(b)** ^13^C NMR spectra in DMSO-d_6_

**a**

**b**

**Figure S11. Merocy-Ald characterization. (a)** ^1^H and **(b)** ^13^C NMR spectra in CDCl_3_.

1. **Movies**
   1. **Movie S1:** *In situ* optical molecular imaging of control lung lobe and *S. aureus* instilled lung lobe during instillation of Merocy-Van, Lung 1.
   2. **Movie S2:** *In situ* optical molecular imaging of control lung lobe and *S. aureus* instilled lung lobe during instillation of Merocy-Van, Lung 2.
   3. **Movie S3:** *In situ* optical molecular imaging of control lung lobe and *S. aureus* instilled lung lobe during instillation of Merocy-Van, Lung 3.

# **References**

(1) Toutchkine, A.; Kraynov, V.; Hahn, K., Solvent-Sensitive Dyes to Report Protein Conformational Changes in Living Cells. *J. Am. Chem. Soc.* **2003,** *125* (14), 4132-4145.

(2) Guan, D.; Chen, F.; Xiong, L.; Tang, F.; Faridoon; Qiu, Y.; Zhang, N.; Gong, L.; Li, J.; Lan, L.; Huang, W., Extra Sugar on Vancomycin: New Analogues for Combating Multidrug-Resistant Staphylococcus aureus and Vancomycin-Resistant Enterococci. *J. Med. Chem.* **2018,** *61* (1), 286-304.

(3) Rossi, A. G.; McCutcheon, J. C.; Roy, N.; Chilvers, E. R.; Haslett, C.; Dransfield, I., Regulation of Macrophage Phagocytosis of Apoptotic Cells by cAMP. *J. Immunol.* **1998,** *160* (7), 3562-3568.
